# Next-Generation Digital Biomarkers for Tuberculosis and Antibiotic Stewardship: Perspective on Novel Molecular Digital Biomarkers in Sweat, Saliva, and Exhaled Breath

**DOI:** 10.2196/25907

**Published:** 2021-08-19

**Authors:** Noe Brasier, Michael Osthoff, Fiorangelo De Ieso, Jens Eckstein

**Affiliations:** 1 Department of Digitalization & ICT University Hospital Basel Basel Switzerland; 2 Institute for Translational Medicine ETH Zurich Zurich Switzerland; 3 Division of Internal Medicine University Hospital Basel Basel Switzerland

**Keywords:** digital biomarkers, active tuberculosis, drug resistance, wearable, smart biosensors, iSudorology, infectious diseases

## Abstract

The internet of health care things enables a remote connection between health care professionals and patients wearing smart biosensors. Wearable smart devices are potentially affordable, sensitive, specific, user-friendly, rapid, robust, lab-independent, and deliverable to the end user for point-of-care testing. The datasets derived from these devices are known as digital biomarkers. They represent a novel patient-centered approach to collecting longitudinal, context-derived health insights. Adding automated, analytical smartphone applications will enable their use in high-, middle-, and low-income countries. So far, digital biomarkers have been focused primarily on accelerometer data and heart rate due to well-established sensors originating from the consumer market. Novel emerging smart biosensors will detect biomarkers (or compounds) independent of a lab and noninvasively in sweat, saliva, and exhaled breath. These molecular digital biomarkers are a promising novel approach to reduce the burden from 2 major infectious diseases with urgent unmet needs: tuberculosis and infections with multidrug resistant pathogens. Active tuberculosis (aTbc) is one of the deadliest diseases from an infectious agent. However, a simple and reliable test for its detection is still missing. Furthermore, inappropriate antimicrobial use leads to the development of antimicrobial resistance, which is associated with high mortality and health care costs. From this perspective, we discuss the innovative approach of a noninvasive and lab-independent collection of novel biomarkers to detect aTbc, which at the same time may additionally serve as a scalable therapeutic drug monitoring approach for antibiotics. These molecular digital biomarkers are next-generation digital biomarkers and have the potential to shape the future of infectious diseases.

## Introduction

A biomarker is defined as “a characteristic that is objectively measured and evaluated as an indicator of normal biological processes, pathogenic processes, or pharmacologic responses to a therapeutic intervention” by the National Institutes of Health Biomarkers Definitions Working Group [1]. Biomarkers are mostly collected from standard biofluids such as blood and urine, among others [2,3]. Compared with unspecific inflammatory markers such as C-reactive protein (CRP), more specific biomarkers such as bacterial culture support diagnosis and disease monitoring [2]. The emergence of smart devices as part of the internet of health care things allows for connecting a patient with health care workers in a location- and lab-independent way [4]. Recording a patient’s health status independent of a lab and remotely provides a novel entity for health and disease data, referred to as a digital biomarker [5]. Smart biosensors collect digital biomarkers such as accelerometer data, heart rate, and body temperature mostly in a noninvasive way. By connecting smart biosensors to automated analytical smartphone applications, digital biomarkers are disrupting standard operating procedures in patient monitoring [6]. The data collected are subsequently stored, shareable, and therefore serve as an outbreak detection network for infectious diseases as described by Tom-Aba et al [7].

Smart biosensors from the consumer market that monitor a patient’s activity, electrocardiogram, heart rate, and other parameters are increasingly available as certified medical devices [8]. The potential to continuously record patient-centered information from daily life will provide deeper insight into health and disease. Investigations into digital biomarkers have been focused on health measurements such as activity and heart rate due to the broad availability of accelerometer sensors. Patients with multiple sclerosis as well as those with Parkinson’s disease–related tremors are subjects of current clinical research [9,10], and studies are also investigating smart device–based automated algorithms to screen for atrial fibrillation using photoplethysmographic rhythm analysis [11].

Point-of-care testing (POCT) provides personalized and actionable case identification near a patient’s location [12]. POCT needs to match the ASSURED criteria according to the World Health Organization (WHO): (1) affordable, (2) sensitive, (3) specific, (4) user-friendly, (5) rapid and robust, (6) equipment-free, and (7) deliverable to the end user [13]. POCT supports health care professionals in high-income countries, as well in middle- and low-income countries with restricted health care infrastructure [14,15]. The most significant barriers for broad use of POCT are lack of training, increased patient waiting time, and lack of availability [16]. Wearable devices linked to automated smartphone applications can be applied at home irrespective of the distance to the nearest health care facility [17]. Novel biosensors will provide the next generation of digital biomarkers by noninvasively detecting molecular feedback [18,19]. The increasingly available smartphone-based biosensors meet the WHO ASSURED criteria and will shape the future of managing infectious diseases around the globe.

From this perspective, we explored novel smartphone-based biosensors to analyze sweat, saliva, and exhaled breath for 2 of the main global issues in infectious diseases. We discuss the potential of molecular digital biomarkers in patients with active tuberculosis (aTbc), 1 of the 3 important epidemics [20]. In addition, we will give a perspective on the use of smart biosensors for noninvasive, personalized, pharmacologic monitoring in patients treated with antibiotics. Antibiotic drug resistance is a threat to global health and a major public health issue [21]. Finally, we will summarize the remaining limitations that have prevented these approaches from being clinically implemented.

## Novel Biosensors for Sweat, Saliva, and Exhaled Breath Analysis

Biofluids such as sweat, saliva, and exhaled breath are noninvasively collectable and represent a promising pool of continuously available molecular biomarkers. So far, sweat, saliva, and exhaled-breath samples have mostly been analyzed using laborious and expensive mass spectrometry [22-24]. Smart biosensors coupled with smartphone applications are increasingly available and allow for lab-independent analysis [25-27]. Smart biosensors connect patients and health care professionals remotely, mostly through Bluetooth or WiFi and may have a global impact in high- as well as in middle- and low-income countries with less available health care infrastructure [4].

### Sweat Sensors

**Figure 1:**
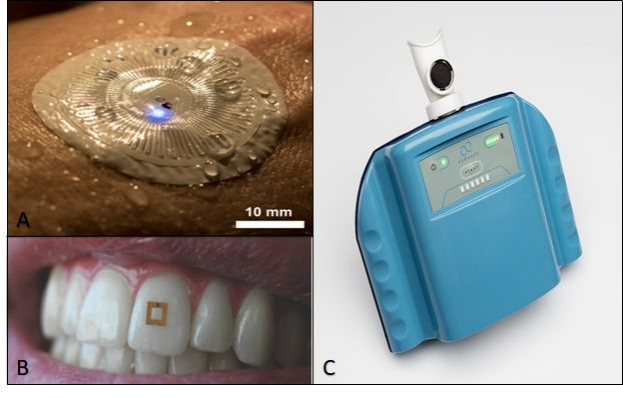
Next–generation biosensors in health care: (A) on-skin sweat biosensor for sweat analysis by Reeder et al [27]; (B) tooth-mounted biosensor for saliva analysis by Tseng et al [25]; and (C) e-nose for exhaled breath analysis by Aeonose [32]. Permission to reproduce the images was obtained from the respective publishers/authors.

Sweat samples are often restricted to low-volume, low-molecule concentrations and need sample stabilization. Noninvasive, lab-independent, on-skin sweat analysis is increasingly available. There are electrochemical-based [28] (Figure 1A), enzyme-linked immunoassay (ELISA)–based [29], and aptameric-based sensors [30], among others. The clinical implementation of sweat biosensors has been restricted, mostly due to unsolved challenges such as sensor stability and low biomarker concentrations. The first clinical tests were successfully conducted using a sweat sensor that detects uric acid as well as tyrosine as described by Yang et al [31].

### Saliva Sensors

A biosensor for in-mouth analysis of saliva has been developed by Tseng et al [25] (Figure 1B). This small sensor is designed to be tooth-mounted and allows for continuous, lab-independent saliva analysis. The functionality of this trilayer sensor was tested in vivo by detecting glucose in different conditions such as dry mouth, after drinking tap water, and after drinking apple juice. Analysis is conducted in combination with a portable vector analyzer attached to a tablet or cell phone. Reliable and stable detection of glucose in vivo was demonstrated for more than 1 week.

### Exhaled Breath Sensors

Biosensors to analyze exhaled breath can be summarized under the term “electric nose” (EN) [26] (Figure 1C). The applicability ranges from health care [33], the food and beverage industry [34], to general monitoring [35]. EN can be compared to the human sense of smell, as the sensor array represents the human nose and data analysis represents the human brain. There are different sensor entities such as low-cost metal oxide sensors performing well at high temperatures; fast and reliable conducting polymer sensors responding to odors; and sensitive, rapid, and stable quartz crystal microbalance [26]. EN has already been clinically validated [36].

### Perspective on Smart Biosensors

Smart biosensors are promising novel approaches to overcome the current remote patient monitoring issues such as lab dependence, laborious sample preparation, and time delays. As analysis can be automated, biosensors will be intuitively easy to handle. The novel opportunity to record continuous molecular feedback will provide novel high-resolution information about health and disease. Nevertheless, easy and continuous health measurements lead to novel issues such as massive amounts of recorded health data and the challenge of interpreting it adequately. Further, due to remaining challenges, such as sensor stability, sample standardization, and detecting low biomarker concentrations, an easy, lab-independent method of detecting complex molecules (eg, proteins) remains an unmet target and requires substantial development and validation efforts. Smart biosensors represent a great potential to solve remaining health care challenges in high-, middle-, and low-income countries. For successful clinical implementation, it will be essential to bring together all stakeholders such as engineers, health care workers, and business partners early, to enable a cost-effective and valuable solution.

## Novel Biomarkers in Sweat, Saliva, and Exhaled Breath to Detect Active Tuberculosis

aTbc is caused by infection with a member of the *Mycobacterium tuberculosis* complex. aTbc is endemic in India, Indonesia, China, Nigeria, Pakistan, and South Africa. From 2000 to 2016, an estimated 53 million people died from tuberculosis infection, and about 10.4 million people were infected per year [20]. To fulfil the WHO End TB Strategy, novel diagnostic markers and devices are urgently needed, as a reliable, straightforward, fast diagnostic test is still lacking [37,38]. Present diagnostic tests are mostly sputum-dependent, which is a significant limitation given that many patients do not produce sputum [39-41]. If sputum cannot be collected spontaneously, an invasive bronchoalveolar lavage is indicated to confirm the diagnosis. Due to limitations of current tests, the patient may remain in contact isolation for weeks until diagnosis. A straightforward test to detect aTbc would enable earlier diagnosis and treatment initiation and therefore potentially lead to a lower transmission rate and decreased morbidity, mortality, and health care costs [42-44].

### Biomarkers in Sweat

By using mass spectrometry analysis, Adewole et al [45] detected 26 specific proteins in the sweat of patients diagnosed with aTbc (Table 1). The researchers were able to differentiate between patients with aTbc, patients with a differential diagnosis such as pneumonia or lung cancer, and healthy controls by taking into account the protein expressions in sweat. Differentiation between the groups was even possible for patients with a history of adequately treated aTbc. The proteomic sweat analysis revealed specific molecules for aTbc such as C1q subcomponent, which has been associated with aTbc [46].

**Table 1:** Overview of next-generation digital biomarkers in sweat, saliva, and exhaled breath for infectious diseases.

Disease or biofluid	In sweat	In saliva	In exhaled breath
Active tuberculosis	Complement C1q subcomponent subunit C, C-reactive protein [45]	Interleukin-1β, interleukin-23, ECM-1, HCC-1, and fibrinogen [47]	Oxetane, 3-(1-methyethyl)-, dodecane, 4-methyl-, cyclohexane, hexyl- [48]
Therapeutic antibiotic drug monitoring	Fluorquinolone (ciprofloxacin), cephalosporines (eg, ceftriaxone) [49]	Cephalosporines (eg, cefuroxime), macrolides (eg, clarithromycin), tetracycline (eg, doxicycline), fluorquinolone (eg, ciprofloxacine) [50]	Carbapeneme (meropenem), acylaminopenicillin (piperacillin) [51]

### Biomarkers in Saliva

Jacobs et al [47] investigated saliva samples from patients presenting with suspected aTbc prior to diagnosis. Patients were subsequently classified using clinical, radiological, and laboratory findings as having aTBc or other respiratory diseases. A 5-biomarker signature detected by Luminex multiplex immunoassay including markers such as interleukin-1β (Table 1) enabled detection of aTbc with a sensitivity of 88.90% and a specificity of 89.75%. Extending the biomarker range led to a sensitivity of 100% and a specificity of 95% (in the absence of HIV) and detected a treatment response from the host after adequate treatment initiation [47].

### Biomarkers in Exhaled Breath

In 1998, Wang et al [52] investigated patients with aTbc and described different levels of nitric oxide in exhaled breath as a potential diagnostic marker using chemiluminescence analysis. By gas chromatographic and mass spectrometric analysis, Phillips et al [48] detected further promising volatile components to differentiate between aTbc and controls (Table 1). Further Nakhleh et al [53] investigated 198 samples from aTbc patients and healthy controls. Of them, 138 samples with known diagnosis were used for unsupervised sensor training, and 60 undefined samples were used for blind validation. In addition, combining exhaled analysis by nanomaterial-based sensors with unsupervised machine learning led to a promising, noninvasive detection tool [54]. Further investigation supported this approach, by using “breathprints” for the detection of *M. tuberculosis* [55].

### Perspective on Digital Biomarkers to Detect aTbc

Detecting and diagnosing aTbc remain laborious and time consuming. Increasing resistance against antimicrobial therapies is further aggravating the burden caused by the disease. As aTbc is mainly endemic in low- and middle-income countries with rather restricted health care access, novel approaches to detect, diagnose, and treat aTbc are needed. An increasing amount of basic research has been conducted recently to define novel aTbc biomarkers. Next to well-known proteins such as C1q and CRP, novel and promising proteins have been explored in sweat, saliva, and exhaled breath of aTbc patients that need further confirmation of value [45]. Smart biosensors for detecting specific aTbc molecules in the aforementioned biofluids have not been applied yet, while e-NOSE has to be considered as an exception [56,57]. Therefore, exhaled breath is the most advanced biofluid when it comes to clinical implementation, while sweat and saliva remain very promising concepts. Nevertheless, exhaled breath may be restricted to pulmonary tuberculosis only. Therefore, sweat and saliva analysis may provide deeper systematic insights into not only pulmonary but also extrapulmonary aTbc.

## Detecting Antibiotics in Sweat, Saliva, and Exhaled Breath for Therapeutic Drug Monitoring

Increasing antimicrobial resistance (AMR) is one of the biggest threats to global health, food security, and development. AMR has a high impact not only on prevention but also on treatment of infectious diseases [58]. Antibiotic misuse and overuse are the main accelerators of the development of AMR [59]. Approximately >30,000 deaths, >870,000 disability-adjusted life years, and >670,000 infections were caused by multidrug resistant organisms in the European Union in 2015 [60]. Antibiotic-resistant bacterial infections lead to a higher rate of complications and a significantly higher need for resources [59]. Improved sanitation, hospital hygiene, infection precautions, and antimicrobial stewardship are proposed solutions to reduce AMR. An important aspect of antimicrobial stewardship is therapeutic drug monitoring (TDM), which is already the standard of care for the treatment with glycopeptides because of their toxicity and TDM’s positive effects on patients’ clinical outcomes [61]. Adult dose recommendations for antibiotics account only barely for renal function, liver function, and weight. Appropriate dosing is crucial, as previous evidence indicates significant interpersonal variability in antibiotic serum concentration and a low chance of achieving even the most conservative pharmacological target [62]. Despite the advances in TDM, a reliable, real-time, noninvasive, and lab-independent monitoring approach is still not available [63,64].

### Antibiotics in Sweat

Høiby et al [49,65] detected ciprofloxacin in sweat in 1997 and certain β-lactam antibiotics in a follow-up study (Table 1) using a microbiological agar diffusion method. Increasing amounts of ciprofloxacin in axillary sweat were associated with the length of antibiotic application time and remained detectable up to 28 hours after the last intake. Further, antibiotic concentrations in sweat may even qualify as a surrogate marker for tissue penetration in soft tissue infections as demonstrated by Brasier et al [66] using ultra high-performance liquid chromatography coupled with quadrupole mass spectrometry. Larger studies that correlate antibiotic concentrations in sweat with clinical outcome are needed to prove this concept.

### Antibiotics in Saliva

Different classes of antibiotics have been detected in saliva so far [50]. Intravenously administered cephalosporins as well as orally administered cephalosporins, tetracycline, and fluoroquinolones are excreted in concentrations above the minimal inhibitory concentration (MIC) in sialadenitis (Table 1) [50,67,68]. However, detected by microbiological agar diffusion, among other methods, phenoxymethylpenicillin was shown to be secreted only in very low concentrations and was therefore deemed not a reasonable treatment approach for sialadenitis [50,67]. Macrolides are an additional class of antibiotics that have been detected in saliva, but in the aforementioned case of sialadenitis, their use has not been indicated due to a high probability of developing antibiotic resistances [69].

### Antibiotics in Exhaled Breath

Herregodts et al [51] recruited 11 critically ill patients from the intensive care unit to investigate the detectability of piperacillin and meropenem in exhaled breath using ultra high-pressure liquid chromatography high-resolution mass spectrometry (Table 1). After collecting breath using the ExaBreath, breath samples were analyzed by mass spectrometry. Piperacillin was detected at a median of 3083 pg/filter (988-203,895 pg/filter) and meropenem at 21,168 pg/filter. However, antibiotic concentrations of 2 of 11 patients were below the lower limit of quantification. Further, antibiotic concentrations in exhaled breath and blood did not correlate. The authors finally concluded that antibiotics in exhaled breath potentially represent the epithelial lining fluid concentrations. More investigations are needed to better understand lung tissue concentrations rather than comparing concentrations in exhaled breath to plasma [51].

### Perspective on Digital Biomarkers for TDM

For sweat analysis, it is unclear if antibiotic concentrations in sweat correlate with the concentrations in blood. However, sweat concentrations may serve as a surrogate marker for antibiotic tissue penetration. This may allow for targeting the detected bacterial MIC obtained by microbial tissue samples in the tissue [65,70]. TDM in saliva potentially shows a comparable weakness, as correlating antibiotic concentrations in saliva and blood remains too ambitious of a target. Still, monitoring antibiotic concentrations in saliva may allow for ensuring antibiotic concentrations above the MIC in bacterial sialadenitis and provide an achievable target in the short term [50]. For exhaled breath, lung diseases such as bacterial pneumonia or pulmonary tuberculosis with a defined MIC seem to be a further achievable monitoring approach.

Overall, it remains to be determined if antibiotic concentrations in sweat, saliva, and exhaled breath correlate with antibiotic concentrations in blood and may serve as systemic, noninvasive TDM in the future. It is highly intuitive that local antibiotic concentrations can potentially be used as a surrogate marker and support the adequate administration of antibiotic drugs in a foreseeable timeframe.

## Discussion

### Principal Findings

From this perspective, we discussed the potential application of novel smart biosensors in aTbc and antimicrobial multidrug resistance, 2 urgent global issues in infectious diseases with unmet needs. Emerging smart biosensors analyzing sweat, saliva, and exhaled breath are increasingly available. These biosensors enable noninvasive sample collection and provide a remote, lab-independent patient monitoring system if combined with smartphones. Moreover, integrating artificial intelligence into the analysis process is a promising addition as it has the potential to support significantly better disease detection and discrimination [71]. Different sensor approaches for on-skin sweat analysis, tooth-mounted sensors for saliva analysis, and e-Noses for exhaled breath analysis are under development at present [25,26,29]. Several specific molecular markers such as the C1q subunit in sweat and interleukin-1β in saliva were detected in patients with aTbc [45,47], thus opening up a new pool of noninvasive biomarkers. Further, different antimicrobials such as β-lactam antibiotics or tetracycline have been detected in sweat, saliva, or exhaled breath [51,65,72]. AMR is a major global threat, and antibiotic stewardship is a proven approach to reduce the development of bacterial resistance [58,73]. Despite the promising approaches and concepts that are considered for leveraging disease burden, only the e-NOSE has been clinically implemented and has to be seen as an exception in this explorative field [56].

A noninvasive, decentralized, lab-independent tool to monitor health and disease is a promising approach to optimize patient care in the future. However, major challenges, such as the lack of technology readiness levels of biosensors, unknown correlations, and high variability between concentrations in sweat, saliva, exhaled breath, and blood, need to be addressed. Before clinical investigation and implementation, a strict and structured validation in laboratory settings is needed to provide internal biosensor validity and clinically interpretable data. Biosensors have to work in different environmental settings, especially when it comes to endemic areas for aTbc, but for TDM as well. Heretofore, high temperatures and high humidity are main challenges to be addressed concerning sensor stability. While biosensor development is expensive and raising funding remains challenging, intensifying basic clinical research and demonstrating clinical value are key to accelerating targeted biosensor development and validation. Due to the decentralized, lab-independent approach and ubiquitous application, smart biosensors provide a scalable approach to impact the health care of the future.

### Further Limitations

Despite the high potential, a few issues remain and have been keeping biosensors from broad clinical implementation, such as low biomarker concentrations and unstable samples, as discussed earlier. In addition, a variety of methods ranging from microbiological agar diffusion methods to mass spectrometry have been used for biofluid analysis. It remains to be determined if the heterogeneously analyzed biomarkers will be detectable by different smart biosensors, and challenges in biofluid sampling need to be addressed during device development. There are further challenging factors for each biofluid.

#### Sweat

Electrolyte concentrations in sweat are known to be dynamic over time when exercising, and sweat rate is known to change with alterations in body temperature [74]. Further, differences in age, gender, and ethnicity have been described and need to be further investigated [75].

#### Saliva

Several physiological factors influence saliva composition; eating habits as well as oral health have also demonstrated a significant impact [76,77]. Moreover, saliva can be contaminated with blood, leading to concentration changes in oxidative stress markers, for example, such as after brushing teeth [78].

#### Exhaled Breath

Detection of biomarkers in exhaled breath is influenced by different habits. Diet and oral health have proven to challenge breath analytics by varying the components [77,79]. Smoking leads to further changes in the breath pattern [80].

### Conclusion

We see great potential in applying smart biosensors to the detection of next-generation digital biomarkers in infectious diseases and provide the first noninvasive, lab-independent, digital point-of-care diagnostics in the future. Modularly combining sweat, saliva, and breath sensors with additional biosensors to collect accelerometer data, heart rate, and body temperature will enable more context-driven monitoring and will support sample standardization. Moreover, if combined with a GPS tracker, those devices allow instant interpretation and monitoring of the spread of infections independently of health care infrastructure on a global scale. To fully investigate the potential of smartphone-based biosensors, more effort is needed to develop, validate, and make those biosensors globally available.

## Abbreviations

AMR: antimicrobial resistance
aTbc: active tuberculosis
CRP: C-reactive protein
ELISA: enzyme-linked immunoassay
EN: electric nose
MIC: minimal inhibitory concentration
POCT: point-of-care testing
TDM: therapeutic drug monitoring
WHO: World Health Organization
